# HoxA9 binds and represses the *Cebpa* +8 kb enhancer

**DOI:** 10.1371/journal.pone.0217604

**Published:** 2019-05-23

**Authors:** Lei Peng, Hong Guo, Peilin Ma, Yuqing Sun, Lauren Dennison, Peter D. Aplan, Jay L. Hess, Alan D. Friedman

**Affiliations:** 1 Division of Pediatric Oncology, Johns Hopkins University, Baltimore, Maryland, United States of America; 2 Department of Pathology and Laboratory Medicine, Indiana University School of Medicine, Indianapolis, Indiana, United Sates of America; 3 Genetics Branch, Center for Cancer Research, NCI/NIH, Bethesda, Maryland, United States of America; Università degli Studi di Milano, ITALY

## Abstract

C/EBPα plays a key role in specifying myeloid lineage development. HoxA9 is expressed in myeloid progenitors, with its level diminishing during myeloid maturation, and HOXA9 is over-expressed in a majority of acute myeloid leukemia cases, including those expressing NUP98-HOXD13. The objective of this study was to determine whether HoxA9 directly represses *Cebpa* gene expression. We find 4-fold increased *HoxA9* and 5-fold reduced *Cebpa* in marrow common myeloid and LSK progenitors from *Vav*-NUP98-HOXD13 transgenic mice. Conversely, *HoxA9* decreases 5-fold while *Cebpa* increases during granulocytic differentiation of 32Dcl3 myeloid cells. Activation of exogenous HoxA9-ER in 32Dcl3 cells reduces *Cebpa* mRNA even in the presence of cycloheximide, suggesting direct repression. *Cebpa* transcription in murine myeloid cells is regulated by a hematopoietic-specific +37 kb enhancer and by a more widely active +8 kb enhancer. ChIP-Seq analysis of primary myeloid progenitor cells expressing exogenous HoxA9 or HoxA9-ER demonstrates that HoxA9 localizes to both the +8 kb and +37 kb *Cebpa* enhancers. Gel shift analysis demonstrates HoxA9 binding to three consensus sites in the +8 kb enhancer, but no affinity for the single near-consensus site present in the +37 kb enhancer. Activity of a *Cebpa* +8 kb enhancer/promoter-luciferase reporter in 32Dcl3 or MOLM14 myeloid cells is increased ~2-fold by mutation of its three HOXA9-binding sites, suggesting that endogenous HoxA9 represses +8 kb *Cebpa* enhancer activity. In contrast, mutation of five C/EBPα-binding sites in the +8 kb enhancer reduces activity 3-fold. Finally, expression of a +37 kb enhancer/promoter-hCD4 transgene reporter is reduced ~2-fold in marrow common myeloid progenitors when the *Vav-*NUP98-HOXD13 transgene is introduced. Overall, these data support the conclusion that HoxA9 represses *Cebpa* expression, at least in part via inhibition of its +8 kb enhancer, potentially allowing normal myeloid progenitors to maintain immaturity and contributing to the pathogenesis of acute myeloid leukemia associated with increased HOXA9.

## Introduction

Hox proteins are best known to mediate pattern formation during early development, but a subset serve additional functions in adult tissues. HoxA9 is preferentially expressed in myeloid progenitors during hematopoiesis, and its level diminishes during normal myeloid maturation [1–3]. Notably, HOXA9 is over-expressed up to 13-fold in >50% of acute myeloid leukemia (AML) cases, and its increased expression is associated with poor prognosis [4, 5]. Golub et al 1999 found that of 6,187 genes evaluated, *HOXA9* over-expression was most highly correlated with treatment failure. Andreef et al 2009 evaluated 119 adult AML cases and that found 20% and 10% long-term survival amongst patients with intermediate or high levels of *HOXA9*, respectively, compared with 40% survival amongst patients with low-level *HOXA9*; in addition, they noted that patients with low-level *HOXA9* mainly had favorable cytogenetics, i.e. t(15;17), t(8;21), or inv(16). *HOXA9* gene expression has been found to be up-regulated in AML cases as a consequence of gene activation by MLL fusion proteins, NUP98 fusion proteins, CALM-AF10, NPM1c mutation, or decreased EZH2 or ASXL1, each often associated with intermediate- or high-risk cases [6].

Transduction of myeloid progenitors with HoxA9-ER and Meis1 leads to their rapid outgrowth as IL-3-dependent cell lines in the presence of 4-hydroxytamoxifen (4HT), and subsequent inactivation of HoxA9-ER by 4HT withdrawal induces their myeloid differentiation [7, 8]. Myeloid progenitor HoxA9 ChIP-Seq data combined with RNA expression analysis in the setting of active versus inactive HoxA9-ER indicates that HOXA9 contributes to induction of genes that favor proliferation and survival (e.g. *c-Myb*, *c-Myc*, *Cdk6*, *CyclinD1*, *Bcl-2*) and to repression of genes that inhibit proliferation (*Ink4a/b*) or direct differentiation (*Cebpa*) [7–9]. Consistent with these findings, *HOXA9* shRNA-mediated knockdown in AML cells leads to their reduced survival and to upregulation of myeloid differentiation markers [10].

The C/EBPα basic region-leucine zipper transcription factor is required for formation of granulocyte-monocyte progenitors (GMP) from common myeloid progenitors (CMP) and is itself mutated in ~10% of AML cases [11]. In addition to its promoter, the murine *Cebpa* gene is regulated by a conserved hematopoietic-specific enhancer located at +37 kb and by a more widely active enhancer located at +8 kb [12–15]. Herein we present data supporting the conclusion that HoxA9 directly binds and inhibits the activity of the *Cebpa* +8 kb enhancer, strengthening the idea that HoxA9 impairs myeloid differentiation via repression of *Cebpa* gene expression in normal hematopoietic stem and progenitor cells and in poor-risk AML cases.

## Materials and methods

### Ethics statement

This study was carried out in strict accordance with the recommendations in the Guide for the Care and Use of Laboratory Animals of the National Institutes of Health. The protocol (M016M66) was approved by the Johns Hopkins University Animal Care and Use Committee. All efforts were made to minimize suffering. Euthanasia was by carbon dioxide asphyxiation.

### Marrow FACS analysis and flow cytometry

C57BL/6 *Vav*-NUP98-HOXD13 and *Cebpa* +37 kb Enh/Prom-hCD4 transgenic mice were previously described [16, 17]. Marrow was obtained by flushing femurs and tibias with phosphate-buffered saline. GMP, CMP, and Lin^-^Sca-1^+^c-Kit^+^ (LSK) marrow cells were enumerated, after red blood cell lysis with ammonium chloride, using biotin-anti-Lineage Cocktail, PerCP-Cy5.5-streptavidin, APC-anti-c-Kit (2B8), PE-Cy7-anti-Sca-1 (D7, eBioscience), PE-anti-CD16/CD32 (FcγR, 2.4G2), and Brilliant Violet 421-anti-CD34 (RAM34). Human CD4 was detected using FITC-anti-hCD4 (RPA-T4). Antibodies were from Pharmingen unless otherwise specified. Marrow subsets for RNA analysis were obtained after lineage-depletion, using biotin-conjugated B220, Gr-1, CD11b, Ter119, and CD3 mouse Lineage Cocktail (BD Pharmingen), anti-biotin microbeads, MACS columns (Miltenyi Biotec), and antibody staining via a FACS Aria II cell sorter (BD Biosciences).

### Cell culture and transduction

32Dcl3 murine myeloid cells [15] were cultured in Iscove’s modified Dulbecco medium (IMDM) with 10% heat-inactivated fetal bovine serum (HI-FBS) and 1 ng/mL murine IL-3 (Peprotech). To induce granulocytic differentiation they were washed twice with phosphate-buffered saline and placed in IMDM, with 10% HI-FBS and 20 ng/mL human G-CSF (Amgen). MOLM14 human AML cells were cultured in RPMI with 10% HI-FBS. 293T cells were cultured in Dulbecco modified Eagle medium with 10% HI-FBS. An *Eco*R1/*Sal*I segment was transferred from MIPuro to MIG-Hoxa9-ER [7] to generate MIPuro-Hoxa9-ER. MIPuro or MIPuro-Hoxa9-ER were packaged into retroviral particles using pkat2ecopac and 293T cells, as described [12], followed by transduction of 32Dcl3 cells for 48 hrs in the presence of 4 μg/mL Polybrene on 12-well plates coated with 25 mg/mL Retronectin. Pooled transductants were obtained by further culture in the presence of 2 μg/mL puromycin. An MIPuro-Hoxa9-ER subclone was then obtained by limiting dilution.

### RNA analysis and western blotting

RNA from hematopoietic cells was prepared using NucleoSpin RNA II, with use of RNase-free DNase (Machery-Nagel). First strand cDNA was prepared using ImProm-II reverse transcriptase (Promega) and oligodT primer at 42°C for 1 hr. Quantitative PCR was carried out using 5–25 ng of each cDNA using Radiant LoRox SYBR Green supermix (Alkali Scientific). *Hoxa9*, *Cebpa*, and ribosomal subunit *mS16* internal control primers were:

HoxA9-F: 5’-AGAAAAACAACCCAGCGAAG,

HoxA9-R: 5’-GGGTTATTGGGATCGATGG,

Cebpa-F: 5’-TGGACAAGAACAGCAACGAG,

Cebpa-R: 5’-TCACTGGTCAACTCCAGCAC,

Mpo-F: 5’-GCTCCGCCCGCATTCCTTGT,

Mpo-R: 5’-TTGAGCTGTGTGGCCAGCCG,

mS16-F: 5’-CTTGGAGGCTTCATCCACAT, and

mS16-R: 5’-ATATTCGGGTCCGTGTGAAG.

Western blotting for HoxA9-ER, using murine ERα antiserum (Santa Cruz Biotechnology), and for β-actin, using monoclonal antibody AC-15 (Sigma), was carried out as described [12].

### Gel shift assay

293T cells were transiently transfected with 6 μg CMV, 3 μg CMV-HoxA9 (kindly provided by E. Eklund), 3 μg CMV-PBX1a (Addgene), or 6 μg CMV-C/EBPα, or with 3 μg of both CMV-HoxA9 and CMV-PBX1a, in 100 mm dishes using 15 μL Lipofectamine 2000 (Invitrogen). Nuclear extracts were prepared two days later and gel shift assay performed, as described [13]. Oligonucleotide probes containing 5′-GCTA or TCGA overhangs were radio-labeled to similar specific activity with the use of Klenow enzyme and α-P^32^-dCTP. Sense strands of the wild-type (WT) probes used, with binding sites underlined, were as follows:

A9/Pbx consensus: 5’-GCTACACATCAATGATTTACGACAACAGGA,

+37kb Enh A9: 5’-GCTACACATCAGTTATTTATCAGAACAGGA, and

NE-C/EBP: 5’-TCGAGGCCAGGATGGGGCAATACAACCCG.

Additional oligonucleotides used as competitors were:

A9/Pbx M1: 5’-GCTACACATCAATGCGTTACGACAACAGGA,

A9/Pbx M2: 5’- GCTACACATCAATGATGGACGACAACAGGA,

37 kb Mut: 5’-GTACACATCAGTTATGGATCAGAACAGGA,

8 kb A9 site1 WT: 5’-GCTATCCTTGAGTGATTTACAATTTGCAAA,

8 kb A9 site1 Mut: 5’-GCTATCCTTGAGTGATGGACAATTTGCAAA,

8 kb A9 site2 WT: 5’-GCTATGCAAACATGTTTTATTTGATTCCCG,

8 kb A9 site2 Mut: 5’-GCTATGCAAACATGTTGGATTTGATTCCCG,

8 kb A9 site3 WT: 5’-GCTAATTCCCACTGATTTATAGGGAATAAG,

8 kb A9 site3 Mut: 5’-GCTAATTCCCACTGATGGATAGGGAATAAG,

8 kb A9 site4 WT: 5’-GCTATCCACAGGACAGAGCATTTATCCCAGAAC,

8 kb A9 site4 Mut: 5’-GCTATCCACAGGACAGAGCATGGATCCCAGAAC,

NE-C/EBP Mut: 5’-TCGAGGCCAGGATGGGCCTATACAACCCG,

α1: 5’-GATCTGATTTACAATTTGCAAACATGTTTTA

α2: 5’-GATCGTTTTATTTGATTCCCGAGTTCTGCCG,

α3: 5’-GATCGAGTTCTGCCGGGGCAATTACAGTGAC,

α4: 5’-GATCTGCTCGCTGTTAAGAAATGTGTTTG,

α5: 5’-GATCAAATGTGTTTGCCTCACTGTTTTGC,

α6: 5’-GATCACTGTACGCGAAGGCAATTTGTTCCAAA,

α7: 5’-GATCTGTGACCACATTCCCACTGATTTATAGG, and

α8: 5’-GATCCCACTGATTTATAGGGAATAAGCCCTAC.

### Transient transfection

The 514 bp murine *Cebpa* +8 kb enhancer was synthesized (Blue Heron) and positioned upstream of the -720/+125 *Cebpa* promoter and luciferase cDNA to generate reporter plasmid *Cebpa* +8 kb Enh/Prom-Luc. Variants of this enhancer with mutations in HoxA9 consensus site 1 (m1), site 2 (m2), site 3 (m3), or in all three sites (m123), or in C/EBPα consensus sites 4 and 5 (m45), 1, 3, and 7 (m137), or all five of these sites (mx5) were also synthesized and positioned similarly. Mutation of the HoxA9 sites matched those in the mutant gel shift competitors. Mutation of the C/EBPα sites changed 5’-T(T/G)NNGNAA(T/G) to 5’-T(T/G)NNCNTA(T/G) in the C/EBPα-binding motif. 5E6 32Dcl3 cells proliferating in IL-3 were transiently transfected with 5 μg of these luciferase reporter DNAs together with 0.5 μg of CMV-βGal using DEAE-dextran and subjected to luciferase and β-galactosidase assays two days later as described [13]. 5E6 MOLM14 cells were transiently transfected similarly with 0.5 μg luciferase reporter DNAs alone, due to their high background β-galactosidase activity.

### Statistics

Means and standard deviations (SD) are shown. The Student *t* test was used for statistical comparisons.

## Results

### HoxA9 represses *Cebpa* gene expression

**Fig 1:**
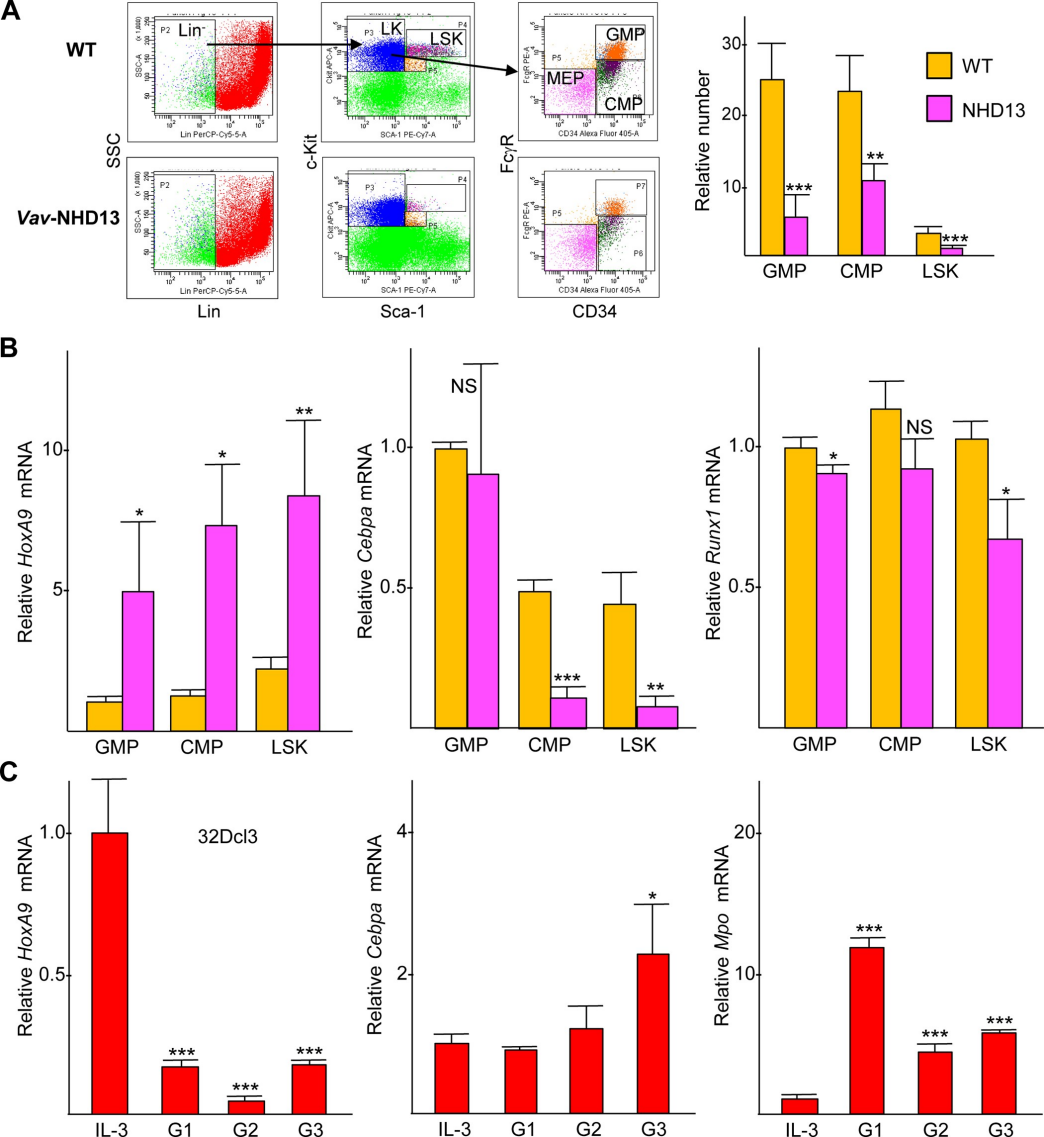
Reciprocal relation between *HoxA9* and *Cebpa* in hematopoietic progenitors. **. A**) GMP, CMP, and LSK cells were enumerated in marrow from 8 wk old wild-type (WT) and *Vav*-NUP98-HOXD13 (*Vav*-NHD13) transgenic mice. Representative FACS analysis (left) and relative absolute number of GMP, CMP, and LSK per hind limbs (right, mean and SD from four mice) are shown. **B)** Total cellular RNAs from GMP, CMP, and LSK cells from WT or *Vav*-NHD13 mice were subjected to quantitative PCR analysis for *HoxA9*, *Cebpa*, and *Runx1* relative to *mS16* ribosomal protein mRNA (mean and SD from three mice). **C)** *HoxA9*, *Cebpa*, and *Mpo* mRNA levels were assessed in 32Dcl3 cells in IL-3 or after transfer to G-CSF for 1, 2, or 3 days (G1, G2, or G3; mean and SD from triplicate analysis in one experiment, with p-values relative to IL-3 level). * p<0.05, ** p<0.01, *** p<0.001, NS—not significant.

Withdrawal of 4HT from murine myeloid progenitors transformed with HoxA9-ER led to ~2-fold increased *Cebpa* mRNA expression at 24 hr and ~3-fold at 48 hr [8]. Pan-hematopoietic expression of the NUP98-HOXD13 (NHD13) myeloid oncoprotein using the *Vav* promoter in mice leads to myelodysplastic syndrome with 4.5-fold increased marrow *HoxA9*, 3.5-fold reduced myeloid CFU-GM colonies, 2-fold reduced LSK stem cells, and 3-fold reduced Lin^-^Sca-1^-^c-Kit^+^ (LK) progenitors [16, 18–20]. Consistent with these results, we find 3-fold reduced GMP, 2-fold reduced CMP, and 6-fold reduced LSK cells in the marrow of 8–10 wk old *Vav*-NHD13 mice (Fig 1A), with 3- to 6-fold increased *HoxA9* mRNA in these marrow subsets (Fig 1B, left). In addition, we find 5-fold reduced *Cebpa* in CMP or LSK cells with only mild reduction in *Runx1* (Fig 1B, center and right), a potential C/EBPα target [13]. Lack of reduced *Cebpa* in GMP may reflect the higher levels of *HoxA9* evident in the CMP and LSK populations. Conversely, when 32Dcl3 murine myeloid cells are induced to differentiate by transfer from IL-3 to G-CSF, *HoxA9* levels decrease and *Cebpa* and *Mpo* levels increase (Fig 1C). These data demonstrate an inverse correlation between *HoxA9* and *Cebpa* in myeloid stem and progenitor cells.

**Fig 2:**
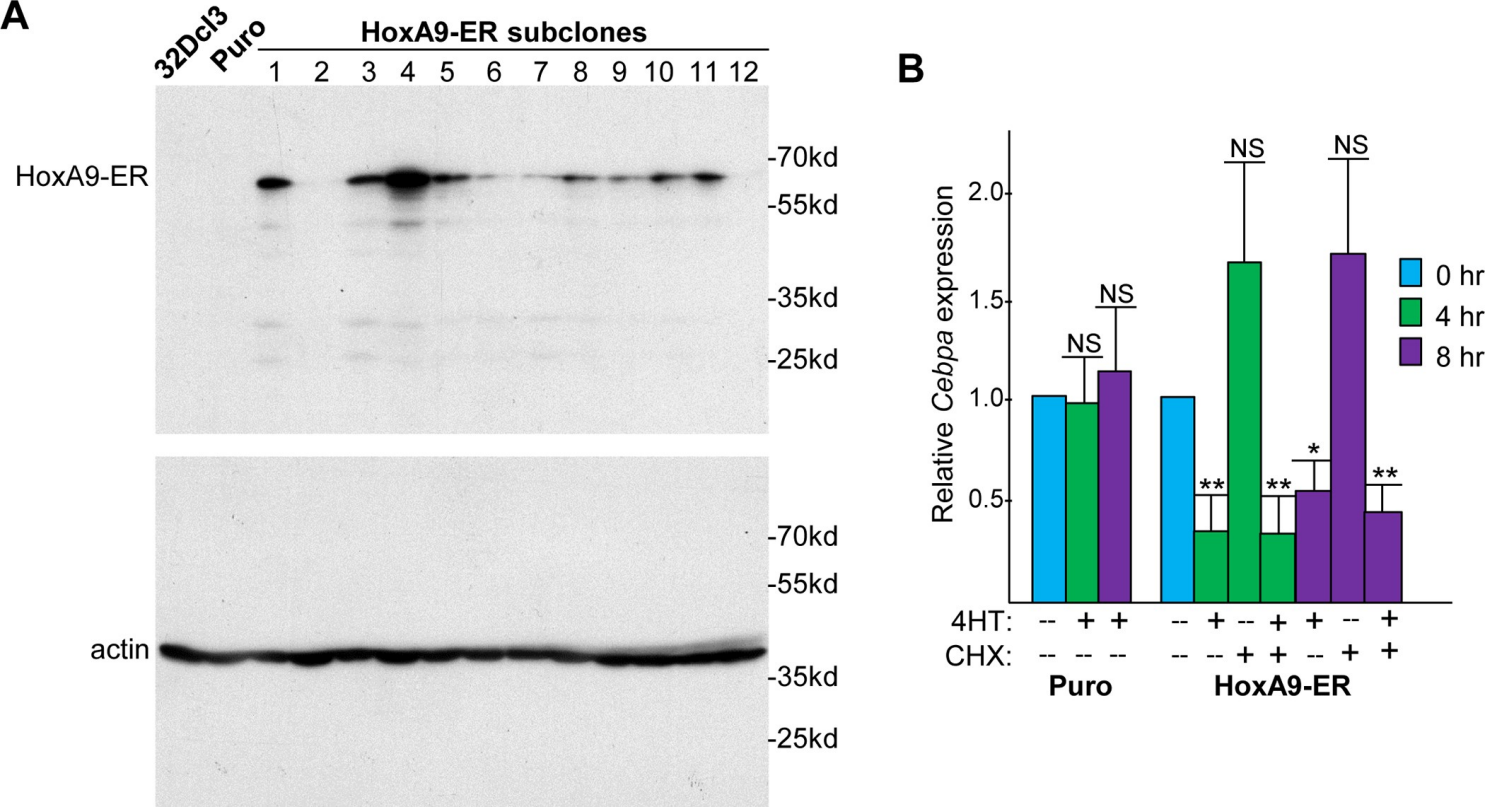
HoxA9-ER directly represses *Cebpa* gene expression in myeloid cells. **A)** Total cellular proteins from parental 32Dcl3 cells, pooled 32Dcl3 MIPuro cells, and twelve 32Dcl3 MIPuro-HoxA9-ER subclones were subjected to Western blotting using anti-rabbit ERα anti-serum, followed by stripping the blot and reprobing with β-actin antibody. **B)** Total cellular RNAs from these cells prepared after 0, 4, or 8 hr of culture in IMDM/HI-FBS/IL-3 media alone, with 200 nM 4-hydroxytamoxifen (4HT), with 50 μg/mL cycloheximide (CHX), or with both were subjected to quantitative PCR analysis for *Cebpa* expression relative to *mS16* mRNA. CHX was added 30 min prior to 4HT when combined (mean and SD from three independent experiments).

In an effort to demonstrate direct repression of *Cebpa* expression by HoxA9, we transduced 32Dcl3 murine myeloid progenitor cells with the MIPuro retroviral vector or with the same vector expressing HoxA9-ER. Western blot analysis detected HoxA9-ER in several 32Dcl3 subclones amongst twelve screened, from which we chose subclone 4 for study (Fig 2A). Note that HoxA9-ER and β-actin are detected at their expected molecular weights of 66 kd and 42 kd, respectively. Activation of HoxA9-ER with 4HT led, on average, to ~3-fold reduction of *Cebpa* mRNA at 4 hr and to ~2-fold reduction at 8 hr, in three independent experiments, with no reduction evident in the MIPuro control cells, and this repression of *Cebpa* mRNA expression was retained in the presence of cycloheximide (CHX), an inhibitor of ribosomal translation (Fig 2B). These findings indicate that HoxA9 directly represses *Cebpa* transcription.

### HoxA9 binds the *Cebpa* +8 kb and +37 kb enhancers

**Fig 3:**
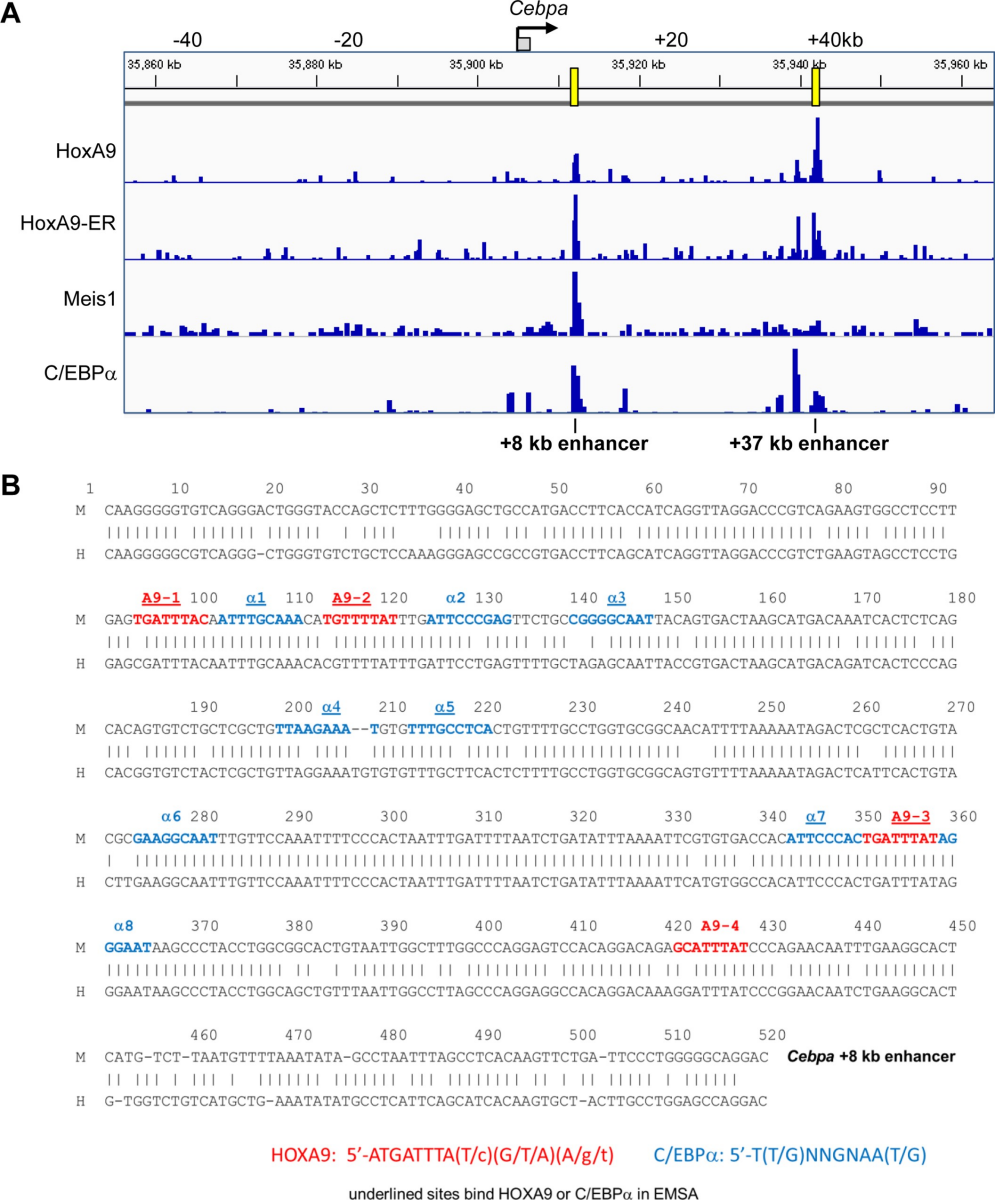
Binding of HoxA9 and C/EBPα within the *Cebpa* locus. **A)** ChIP-Seq data at the *Cebpa* locus for exogenous, HA-tagged HoxA9, HoxA9-ER, and Meis1 and for endogenous C/EBPα in HoxA9/Meis1- or HoxA9-ER/Meis1-transformed murine myeloid progenitors is shown [7, 21]. **B)** The sequence of the murine (M) *Cebpa* +8 kb enhancer is aligned with the corresponding human (H) + 9 kb *CEBPA* enhancer. Positions of sites that match or nearly match the HoxA9 or C/EBPα consensus-binding motifs are shown, with those underlined found in this report to bind these proteins in electrophoretic mobility shift assay (EMSA).

ChIP-Seq data for HA-HoxA9, HA-HoxA9-ER, and HA-Meis1, obtained using anti-HA antibody, or for endogenous C/EBPα [7, 21], demonstrates that HoxA9 and C/EBPα interact with both the +8 kb and +37 kb *Cebpa* enhancers, whereas Meis1 binds exclusively to the +8 kb enhancer (Fig 3A). Interaction of C/EBPα with both enhancers is consistent with ChIP-Seq data obtained using murine GMP [13]. In contrast, Runx1, PU.1, GATA-2, and SCL bind exclusively within the +37 kb enhancer [13].

HoxA9 binds DNA as a HoxA9:Pbx dimer or as a HoxA9:Pbx:Meis1 trimer [22]. PCR-mediated site selection identified 5’-ATGATTTACGAC as the optimal HoxA9:Pbx1 binding site [23]. Within this sequence, TGA is thought to bind Pbx and TTTACGAC HoxA9, based on their co-crystal structure [24]. Analysis of *in vivo* binding sites identifies 5’-TTTA(T/c)(G/T/A)(A/g/t) as preferred [25]. Based on these consensus sequences, we identified potential HoxA9-binding sites within the *Cebpa* +8 kb and +37 kb enhancers. The +37 kb enhancer contains the sequence 5’-GTTATTTATCA, which differs from the consensus at the underlined three positions. Of note, this potential HoxA9-binding site overlaps with 5’-CAGTTA, which binds c-Myb, and with 5’-TGATAA on the opposite DNA strand, which binds GATA-2 in gel shift assays [13]. The +8 kb enhancer contains four potential HoxA9-binding sites (Fig 3B). Site 1, 5’-TGATTTACAA, perfectly matches the consensus. Site 2, 5’-TGTTTTATTT, has one mismatch in the Pbx half-site. Site 3, 5’-TGATTTATAG, perfectly matches the consensus. Site 4, 5’-GCATTTATCC, has four mismatches.

**Fig 4:**
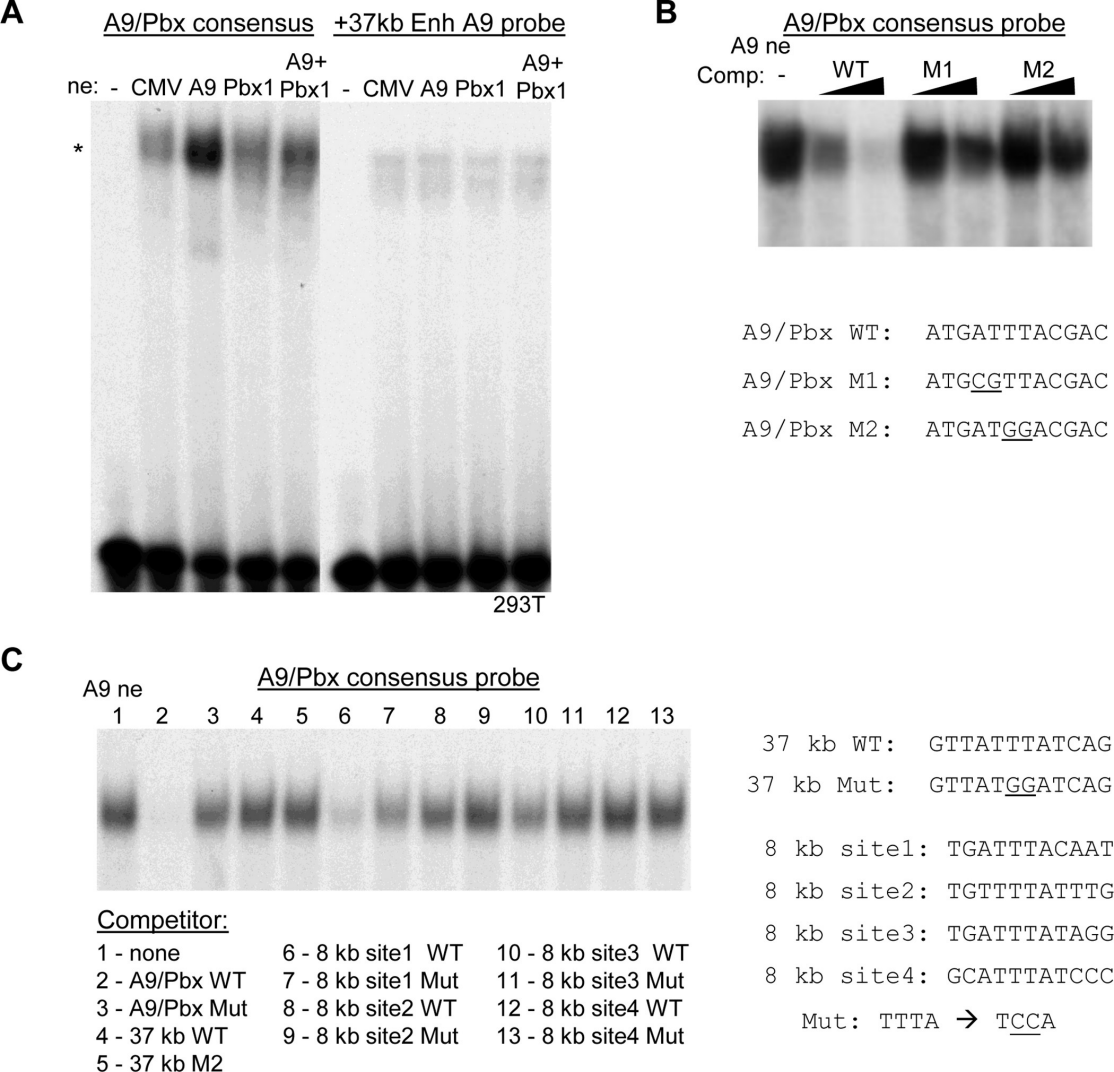
HoxA9 binds three sites within the *Cebpa* +8 kb enhancer. **A)** 1 ng of radio-labeled HoxA9 (A9)/Pbx consensus probe or *Cebpa* +37 kb enhancer HoxA9-binding site probe was incubated with 10 μg of nuclear extract prepared from 293T cells transfected with empty CMV vector or vectors expressing HoxA9, Pbx1, or both, followed by non-denaturing polyacrylamide gel electrophoresis (PAGE) and autoradiography. The position of the shifted species is indicated (*). **B)** 1 ng of radio-labeled A9/Pbx consensus probe was incubated with HoxA9-expressing nuclear extract alone or in the presence of 5 ng or 25 ng of unlabeled wild-type (WT) probe or either of two mutant probes (M1, M2), followed by PAGE and autoradiography. The sequences of the HoxA9-binding motifs within the WT and mutant A9/Pbx probe are shown, with mutant bases underlined. **C)** 1 ng of radio-labeled A9/Pbx consensus probe was incubated with HoxA9-expressing nuclear extract alone or in the presence of 25 ng of the indicated competitors, followed by PAGE and autoradiography. The HoxA9 motifs within the *Cebpa* +8 kb enhancer DNAs are shown, along with the base changes present in the mutant (Mut) competitors.

293T nuclear extracts, prepared after transfection with empty CMV vector, CMV-HoxA9 (A9), CMV-PBX1a (Pbx1), or both CMV-HoxA9 and CMV-PBX1a, were subject to gel shift analysis using radio-labelled HoxA9/Pbx (A9/Pbx) consensus or +37 kb enhancer HoxA9 site probes (Fig 4A). Weak binding to both probes was evident using the empty CMV nuclear extract, indicative of endogenous protein(s) capable of interacting with each probe. Increased binding to the consensus probe, but not to the +37 kb enhancer probe, was evident using the A9 or A9+Pbx1 extracts. Binding to the consensus probe was stronger using the A9 compared with the A9+Pbx1 extract, which may reflect sufficient endogenous Pbx and CMV promoter competition reducing HoxA9 expression when the two plasmids are co-transfected. The HoxA9 nuclear extract was then subjected to gel shift analysis using the radio-labelled A9/Pbx consensus probe in the presence of no competitor, 5- or 25-fold excess of unlabeled WT probe, or 5- or 25-fold excess of either of two mutant oligonucleotides having 2 bp point mutations in the consensus HoxA9-binding site (Fig 4B). The WT probe was more effective than the mutant probes at competing for HoxA9 binding, as shown by decreased signal in the presence of excess unlabeled WT probe compared to the mutant probes, indicating binding of HoxA9 to the predicted binding motif.

Finally, the A9/Pbx consensus probe was subject to gel shift assay using the A9 nuclear extract in the presence of no competitor, 25-fold excess of WT or mutant A9/Pbx consensus probe, WT or mutant +37 kb enhancer probe, or WT or mutant HoxA9-binding sites 1–4 from the +8 kb enhancer (Fig 4C). Again, WT but not mutant A9/Pbx probe competed effectively (lanes 2, 3). The +37 kb enhancer WT probe was ineffective as a competitor (lane 4), consistent with the lack of evident binding above background when this probe was radio-labeled and combined with exogenous HoxA9. +8 kb enhancer WT sites 1, 2, and 3, but not 4, competed for binding of HoxA9 to the consensus probe (lanes 6, 8, 10, and 12), with mutation of sites 1, 2, or 3 reducing competition (lanes 7, 9, and 11). Of these four sites, site 1 was the most effective competitor, followed by site 3, reflecting their fully matching the 5’-TTTA(T/c)(G/T/A)(A/g/t) consensus. Lack of affinity to site 4 is likely due to its four mismatches with this consensus, compared with site 2 which has only one mismatch. The lack of affinity seen when the +37 kb enhancer site was used as a probe, and its inability to act as competitor under these conditions, likely reflects its three mismatches from the HoxA9 consensus. Overall, these data indicate that HoxA9 binds directly to the +8 kb *Cebpa* enhancer via three sites and that its interaction with the +37 kb enhancer evident in myeloid progenitor ChIP-Seq may reflect indirect contact via one or more bound transcription factors.

### C/EBPα binds the *Cebpa* +8 kb enhancer

**Fig 5:**
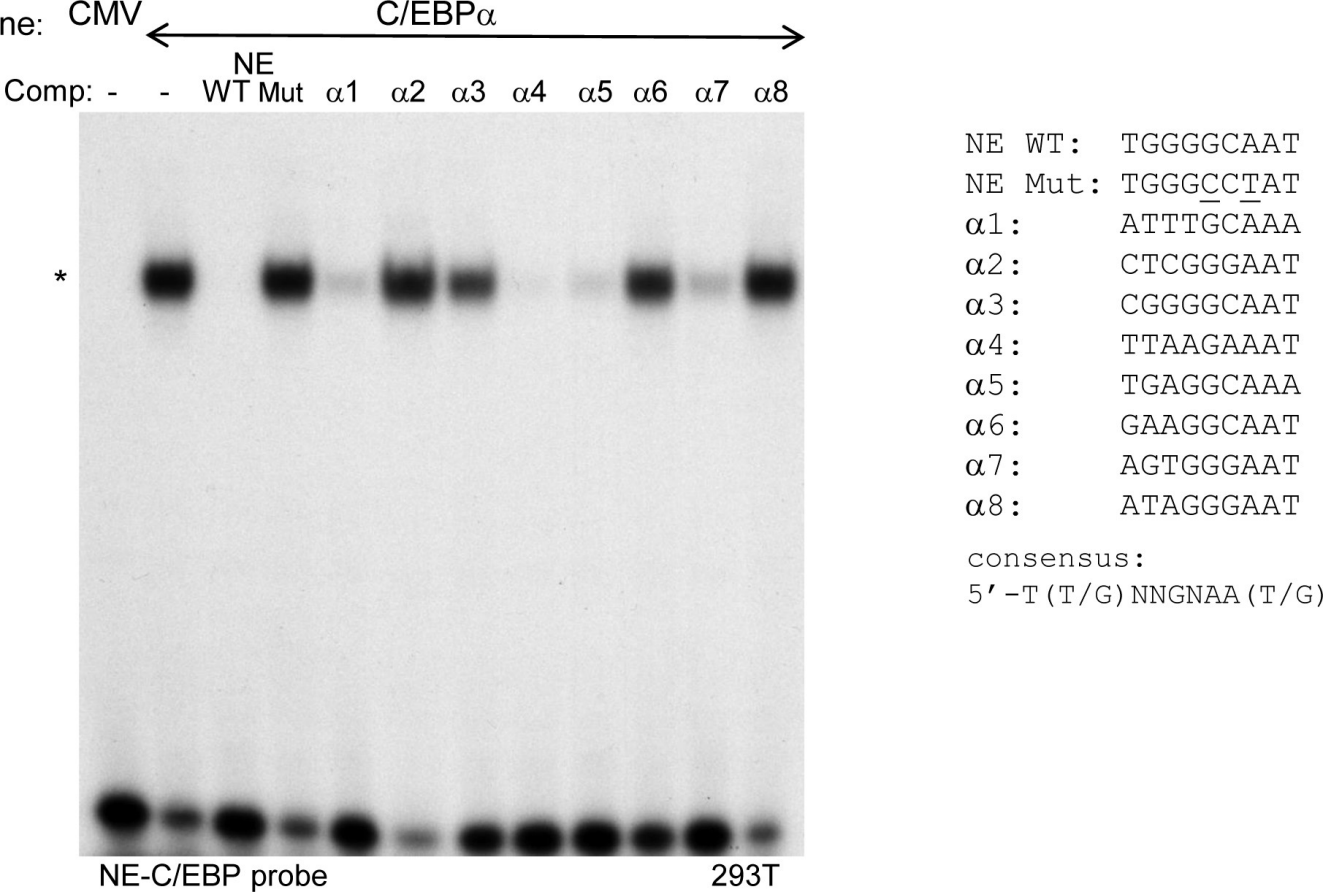
C/EBPα binds five sites within the *Cebpa* +8 kb enhancer. 1 ng of radio-labeled NE-C/EBP probe, containing a C/EBPα-binding site, was incubated with 10 μg of nuclear extract prepared from 293T cells transfected with empty CMV vector or with a vector expressing C/EBPα, alone or with 25 ng of the indicated unlabeled competitor double-stranded DNAs, followed by PAGE and autoradiography. The position of the shifted species is indicated (*). The sequences of the C/EBP-binding motifs within the competitors are shown with mutant bases underlined.

Binding motifs for C/EBP, RUNX1, PU.1, and MYB family transcription factors are commonly found in the vicinity of HoxA9 binding sites identified via ChIP-Seq in myeloid progenitors [7–9]. While RUNX1 and PU.1 interact with the +37 kb *Cebpa* enhancer, neither interacts with the +8 kb enhancer [13]. In addition, c-Myb did not bind either enhancer when assessed by ChIP-Seq using a murine myeloid progenitor cell line [26]. C/EBPα binds two sites within the *Cebpa* +37 kb enhancer, and mutation of these sites reduces enhancer activity in 32Dcl3 myeloid cells [13]. In addition, we identified eight sequences within the +8 kb enhancer that matched or nearly matched the C/EBP-binding consensus (Fig 3B). Gel shift analysis was conducted using a radio-labeled probe containing a known C/EBPα-binding site in the neutrophil elastase (NE) promoter and nuclear extract prepared from 293T cells expressing exogenous C/EBPα, together with no competitor, 25-fold excess of unlabeled WT or mutant NE-C/EBP probe, or 25-fold excess of unlabeled double-stranded oligonucleotides containing each of the candidate C/EBPα-binding sites (Fig 5). Strong competition was seen with WT but not mutant NE-C/EBP. In addition, enhancer sites 1, 4, 5, and 7 competed strongly and site 3 competed modestly with the NE probe for C/EBPα binding. Thus, C/EBPα interacts with five *Cebpa* +8 kb enhancer sites *in vitro*, consistent with its *in vivo* interaction with the enhancer in myeloid progenitors.

### Endogenous HoxA proteins repress and C/EBP proteins activate the +8 kb enhancer

**Fig 6:**
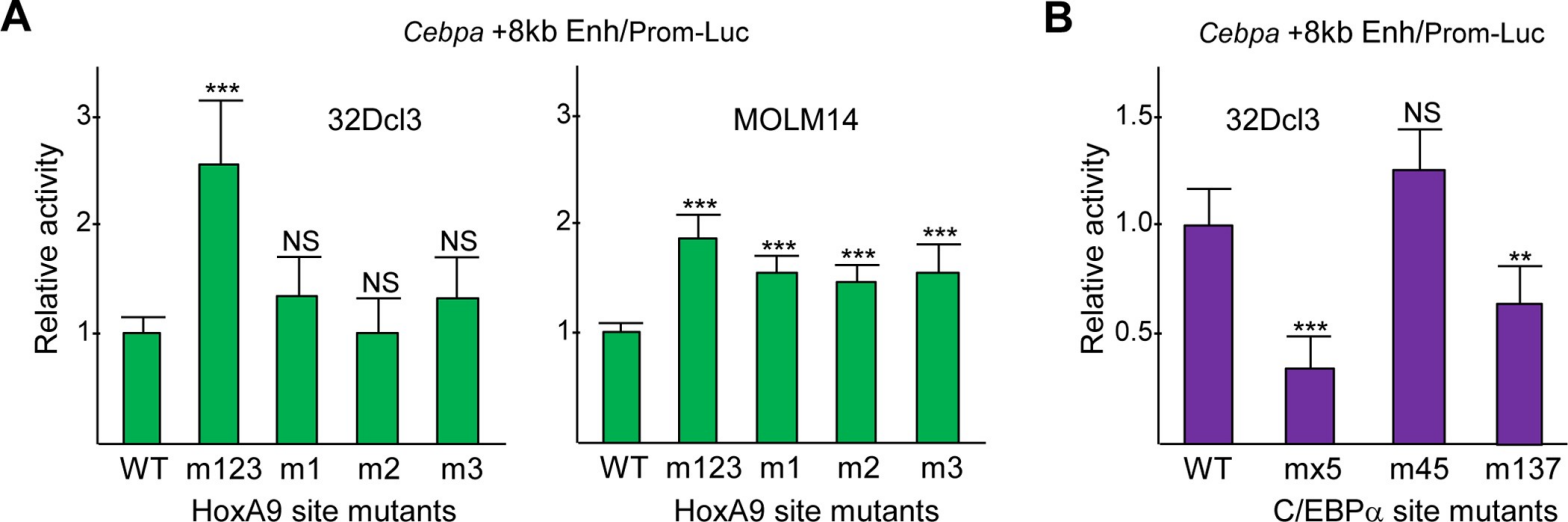
HoxA9 represses and C/EBPα activates the *Cebpa* +8 kb enhancer in immature myeloid cells. **A)** 32Dcl3 murine myeloid progenitor or MOLM14 human AML cells were transfected with wild-type (WT) *Cebpa* +8 kb Enh/Prom-Luc or with mutant variants harboring 2 bp point mutations in HoxA9-binding sites 1, 2, and 3 (m123), or in sites 1, 2, or 3 alone (m1, m2, m3), together with CMV-βGal as internal control for 32Dcl3 cells. The relative activity of each reporter is shown, with the activity of the WT reporter set to 1.0 in each experiment (mean and SD from six determinations for 32Dcl3 and nine for MOLM14). **B)** 32Dcl3 cells were transfected with WT *Cebpa* +8 kb Enh/Prom-Luc or with mutant variants harboring 2 bp point mutations in C/EBP**α**-binding sites 1, 3, 4, 5, and 7 (mx5), 4 and 5 (m45), or 1, 3, and 7 (m137), with CMV-βGal. The relative activity of each reporter is shown, with the activity of WT reporter set to 1.0 in each experiment (mean and SD from nine determinations).

Point mutations were introduced into HoxA9-binding sites 1–3, or into each site individually, within the 514 bp +8 kb *Cebpa* enhancer, and these variants or the WT enhancer were then positioned upstream of the -720/+125 bp *Cebpa* promoter and the luciferase cDNA to generate reporter constructs. *Cebpa* +8 kb Enh/Prom-Luc or its mutant variants were transiently transfected into murine 32Dcl3 myeloid cells along with CMV-βGal, followed by assessment of luciferase activity 48 hr later, normalized to β-galactosidase activity (Fig 6A, left). Mutation of all three HoxA9 sites increased activity 2.5-fold, on average. Mutation of each site individually mildly increased reporter activity, but not to a significant degree. These same luciferase constructs were also transiently transfected into human MOLM14 AML cells, without CMV-βGal due to high endogenous β-galactosidase activity. MOLM14 cells express the MLL-AF9 fusion oncoprotein, an inducer of *HOXA9* transcription [10]. Mutation of the three HoxA9-binding sites again increased reporter activity in this context, 1.8-fold on average, and mutation of each individual binding site also increased activity, although to a lesser extent (Fig 6A, right). These data indicate that endogenous HoxA transcription factors repress *Cebpa* +8 kb enhancer activity in immature myeloid progenitor or leukemia cells.

Point mutations were also introduced into C/EBP**α**-binding sites 1, 3, 4, 5, and 7, into adjacent sites 4 and 5, or into sites 1, 3, and, 7 within +8 kb *Cebpa* enhancer, and these variant enhancers were positioned upstream of the *Cebpa* promoter and luciferase cDNA. *Cebpa* +8 kb Enh/Prom-Luc or its variants were transiently transfected into murine 32Dcl3 myeloid cells along with CMV-βGal, followed by assessment of luciferase activity 48 hr later, normalized to β-galactosidase activity (Fig 6B). Mutation of all five C/EBP**α**-binding sites reduced reporter activity 3-fold, on average. Mutation of sites 4 and 5 had no effect, while mutation of sites 1, 3, and 7 reduced activity ~2-fold. These data indicate that endogenous C/EBP proteins activate the *Cebpa* +8 kb enhancer in 32Dcl3 myeloid cells.

### +37 kb enhancer/promoter activity is reduced in the context of elevated HoxA9

**Fig 7:**
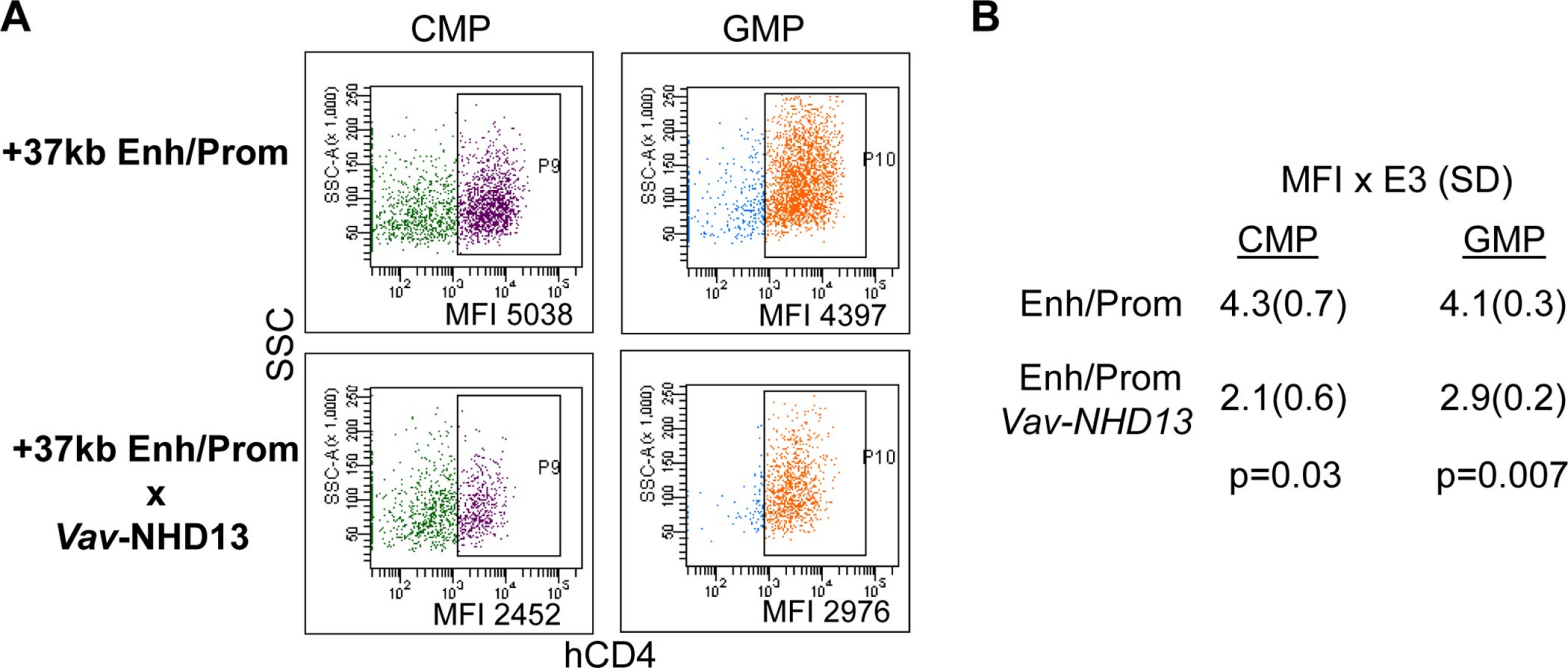
Expression of a *Cebpa* +37 kb enhancer/promoter transgene is reduced in hematopoietic progenitors with increased HoxA9. **A)** Representative flow cytometry showing expression of the *Cebpa* +37 kb enhancer/promoter (Enh/Prom)-human CD4 (hCD4) transgene after breeding to introduce one *Vav*-NUP98-HOXD13 (NHD13) allele. SSC—side scatter. **B)** The geometric mean fluorescence intensity (MFI) for hCD4 within CMP or GMP expressing the transgene is shown (mean and SD from three determinations).

As the near consensus HoxA9 site in the +37 kb enhancer did not interact with HoxA9 *in vitro* and overlaps with motifs that bind GATA-2 and c-Myb, we did not evaluate the effect of its mutation on +37 kb enhancer activity. However, we did take advantage of transgenic mice harboring a +37 kb *Cebpa* enhancer/promoter-hCD4 reporter that expresses cytoplasmically truncated, plasma membrane hCD4 at high levels in CMP and GMP [17]. These mice were bred to *Vav*-NHD13 mice, which have elevated HoxA9 in these marrow subsets. Compared with progenitors from *Cebpa* +37 kb enhancer/promoter mice, introduction of the NHD13 transgene reduces hCD4 geometric mean fluorescence intensity (MFI) 2.1-fold, on average, in CMP and 1.4-fold in GMP (Fig 7). These findings, coupled with ChIP-Seq data showing that HoxA9 binds the *Cebpa* +37 kb enhancer but not its promoter, suggest that HoxA9 also represses *Cebpa* transcription via its +37 kb enhancer.

## Discussion

C/EBPα is required for normal myeloid development, and reduced C/EBPα expression or activity is evident in the majority of AML cases [11]. The murine *Cebpa* gene contains a 455 bp enhancer located at +37 kb that is highly conserved in the human *CEBPA* locus and functions specifically in hematopoietic stem and progenitor cells [12–15]. The +37 kb enhancer is bound and activated by RUNX1, C/EBPα, GATA-2, SCL, NR4A1/3, PU.1, additional ETS family transcription factors, and potentially c-Myb in normal myeloid progenitors and is bound and potentially repressed by RUNX1-ETO in AML cases [13, 27, 28]. Analysis of H3K4me1 and H3K27Ac histone marks in murine GMP, CMP, and additional marrow hematopoietic progenitors identified a second potential enhancer located at +8 kb in the *Cebpa* locus [13]. Similar analysis of H3K27Ac marks in the human *CEBPA* locus indicates that the corresponding +9 kb enhancer is active in myeloid cells and also in all non-myeloid, C/EBPα-expressing tissues examined, i.e. liver, lung, adipose, large and small intestines, skin epithelium, and placenta; moreover, 4C-Seq analysis demonstrates interaction of the +9 kb enhancer with the *CEBPA* promoter in both myeloid and non-myeloid cell lines [15]. Our prior analysis of ChIP-Seq data indicates that C/EBPα, but not RUNX1, PU.1, GATA-2, or SCL, binds the +8 kb enhancer [13]. We now further demonstrate that HoxA9 directly binds the +8 kb *Cebpa* enhancer via three binding sites and that HoxA9 also localizes to the +37 kb enhancer, likely via interaction with other bound transcription factors. We show that increased HoxA9 resulting from NHD13 myeloid oncoprotein expression is associated with reduced *Cebpa* in CMP and LSK marrow cells, decreased HoxA9 as 32Dcl3 cells differentiate is associated with increased *Cebpa*, activation of exogenous HoxA9-ER reduces *Cebpa* even in the presence of a ribosomal translation inhibitor, and mutation of the three HoxA9-binding sites in the +8 kb enhancer increases reporter activity in two myeloid cell lines. Moreover, inactivation of HoxA9-ER in myeloid progenitors leads to increased *Cebpa* mRNA [8]. Together, these data indicate that HoxA9 binds and represses *Cebpa* +8 kb enhancer activity.

Within hematopoiesis HoxA9 expression is prominent in hematopoietic stem and myeloid progenitors and diminishes during myeloid maturation [1–3]. Our findings suggest that reduced HoxA9 is a prerequisite for *Cebpa* to achieve a level required for granulocytic and monocytic differentiation. HOXA9 expression is also prominent in >50% of human AML cases [4–6], where it might contribute to myeloid transformation in part by reducing *CEBPA* expression and thereby interfering with myeloid differentiation. In addition, our finding that C/EBPα plays a role in early B lymphopoiesis suggests that HOXA9 induction by MLL-AF4 or other MLL fusion oncoproteins in preB ALL cases might also suppress *CEBPA* expression to contribute to leukemic transformation [6, 29].

HoxA9 is co-expressed with additional HoxA proteins, in particular HoxA5, HoxA7, and HoxA10, in immature hematopoietic populations, which likely accounts for the observation that *HoxA9*-/- mice manifest only mild pancytopenia [30]. Expression of the corresponding human *HOXA* genes are often increased together with *HOXA9* in human AMLs [1–6]. As the HOXA proteins have similar DNA-binding consensus motifs [22–24], the increased activity of the *Cebpa* +8 kb enhancer/promoter luciferase reporter seen in 32Dcl3 and MOLM14 cells upon mutation of the three enhancer HoxA9-binding sites may in part reflect reduced binding of not only HoxA9 but also additional HoxA proteins. Of note, while Pbx antibody super-shifted the entire HoxA9 consensus complex formed in gel shift assay with 32Dcl3 nuclear extract, HoxA9 antibody only shifted ~25% of this complex, indicating the presence of additional HoxA proteins [31]. Nevertheless, as HOXA9 is most consistently induced in murine and human AMLs and correlates most highly with poor prognosis [4–6], as absence of HoxA9 prevents transformation by MLL-ENL [32], and as HOXA9 depletion is sufficient to induce proliferation arrest and apoptosis in AML cell lines and in primary AML cells [10], HOXA9 may be the HOXA transcription factor most critical for normal and malignant myelopoiesis.

In addition to repression by HoxA9, we also provide data indicating that C/EBPα directly binds and activates the *Cebpa* +8 kb enhancer via five consensus sites, as mutation of these five binding sites leads to increased *Cebpa* +8 kb enhancer/promoter activity in 32Dcl3 cells. C/EBPα was previously shown to bind and activate the *Cebpa* promoter and +37 kb enhancer [13, 33]. Additional C/EBP proteins may also activate the +8 kb enhancer, although C/EBPα is the most prominent isoform in immature myeloid cells, including in 32Dcl3 cells where C/EBPα represents the large majority and C/EBPβ only a minor proportion of gel shift species evident using the consensus NE-C/EBP probe [34, 35].

Enhancers bound by HoxA9 in myeloid progenitors often harbor C/EBP motifs [7–9], and HoxA9 and C/EBPα directly interact [21]. Whether this interaction facilitates cooperative DNA-binding and/or engenders cooperative or cross-inhibitory trans-activation activity at these enhancers requires future investigation. Notably, within the *Cebpa* +8 kb enhancer, the three HoxA9-binding sites are located directly adjacent to C/EBPα-binding sites, raising the possibility that HoxA9 and C/EBPα compete for binding to this enhancer. Of note, among non-hematopoietic tissues that express C/EBPα, several also express *HoxA9*, i.e. adipose, small and large intestine, and skin, but not liver, lung, or placenta (https://gtexportal.org), potentially reflecting their mutual regulation of the *Cebpa* +8 kb and additional enhancers also in these tissues.

Simultaneous binding of HoxA9 and C/EBPα to the +8 kb enhancer in myeloid progenitors may generate a primed transcriptional state which is resolved upon decrease in HoxA9 during myeloid maturation. Consistent with this idea, the enhancer is strongly marked by H3K4me1 but only very weakly by H3K27Ac and not at all by H3K27me3 in HoxA9/Meis1-immortalized myeloid progenitors (S1 Fig). A primed pattern is also evident at the +8 kb enhancer in GMP, with the activating H3K27Ac histone mark increased substantially in granulocytes [13], potentially reflecting absence of HoxA9 at this terminal stage of myeloid differentiation.

Finally, our finding that a *Cebpa* +37 kb enhancer/promoter-human CD4 transgene has reduced activity in CMP and GMP when HoxA9 levels are elevated by a *Vav*-NHD13 transgene supports the possibility that interaction of HoxA9 with the +37 kb enhancer may also contribute to repression of *Cebpa* gene expression during early hematopoiesis or myeloid transformation.

## Supporting information

S1 Fig The *Cebpa* +8 kb enhancer is primed in HoxA9/Meis1-immortalized myeloid progenitors. ChIP-Seq data for C/EBPα, HoxA9, H3K27me3, H3K27Ac, and H3K4me1 at the murine *Cebpa* locus [9, 21].
(TIF)
