# Supplementary figures and images for: HoxA9 binds and represses the Cebpa +8 kb enhancer
Source: PLoS One. 2019 May 23;14(5):e0217604. doi: 10.1371/journal.pone.0217604 (PMC6532930; doi:10.1371/journal.pone.0217604)

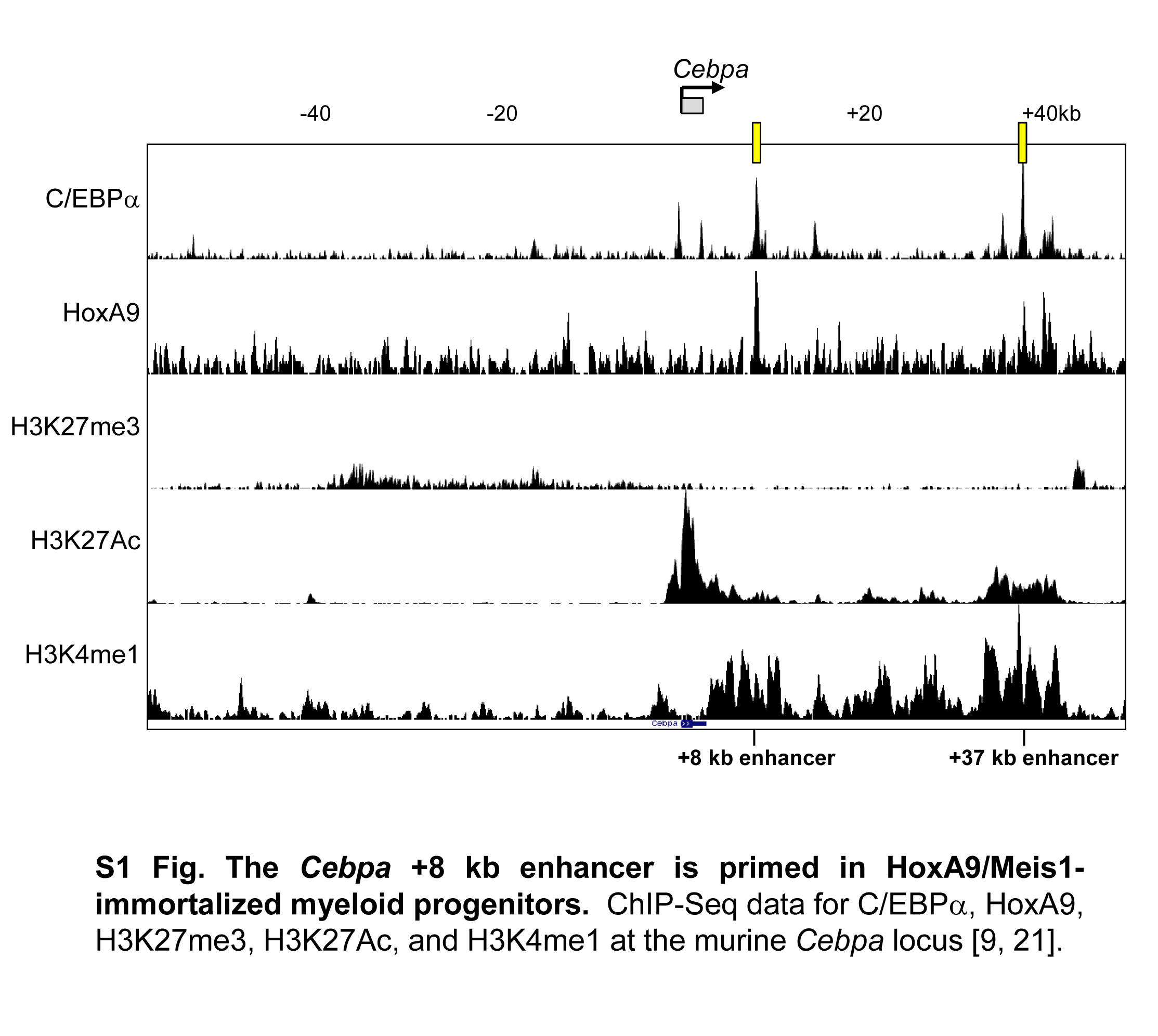

Supplement: S1 Fig — ChIP-Seq data for C/EBPα, HoxA9, H3K27me3, H3K27Ac, and H3K4me1 at the murine Cebpa locus [9, 21]. (TIF) [file pone.0217604.s001.tif]
